# The role of [^68^ Ga]Ga-DOTATATE PET/CT in wild-type *KIT*/*PDGFRA* gastrointestinal stromal tumours (GIST)

**DOI:** 10.1186/s13550-021-00747-0

**Published:** 2021-01-14

**Authors:** Luigi Aloj, Olivier Giger, Iosif A. Mendichovszky, Ben G. Challis, Meytar Ronel, Ines Harper, Heok Cheow, Rogier ten Hoopen, Deborah Pitfield, Ferdia A. Gallagher, Bala Attili, Mary McLean, Robin L. Jones, Palma Dileo, Venkata Ramesh Bulusu, Eamonn R. Maher, Ruth T. Casey

**Affiliations:** 1 Department of Radiology, University of Cambridge, Cambridge, CB2 0QQ UK; 2 Department of Nuclear Medicine, Cambridge University Hospitals Foundation Trust, Cambridge, CB2 0QQ UK; 3 Department of Pathology, University of Cambridge, Cambridge, CB2 0QQ UK; 4 Department of Endocrinology, Cambridge University Hospitals Foundation Trust, Cambridge, CB2 0QQ UK; 5 Department of Oncology, University of Cambridge, Cambridge, CB2 0QQ UK; 6 Cancer Research UK Cambridge Centre, Cambridge, UK; 7 Department of Medical Oncology, Royal Marsden NHS Foundation Trust and Institute of Cancer Research, London, SW3 6JJ UK; 8 Department of Medical Oncology, University College London Hospital Foundation Trust, London, NW1 2PG UK; 9 Department of Medical Oncology, Cambridge University Hospitals Foundation Trust, Cambridge, CB2 0QQ UK; 10 Department of Medical Genetics, University of Cambridge and NIHR Cambridge Biomedical Research Centre and Cancer Research UK Cambridge Centre, Cambridge, CB2 OQQ UK

## Abstract

**Background:**

[^68^ Ga]Ga-DOTATATE PET/CT is now recognised as the most sensitive functional imaging modality for the diagnosis of well-differentiated neuroendocrine tumours (NET) and can inform treatment with peptide receptor radionuclide therapy with [^177^Lu]Lu-DOTATATE. However, somatostatin receptor (SSTR) expression is not unique to NET, and therefore, [^68^ Ga]Ga-DOTATATE PET/CT may have oncological application in other tumours. Molecular profiling of gastrointestinal stromal tumours that lack activating somatic mutations in *KIT* or *PDGFRA* or so-called ‘wild-type’ GIST (wtGIST) has demonstrated that wtGIST and NET have overlapping molecular features and has encouraged exploration of shared therapeutic targets, due to a lack of effective therapies currently available for metastatic wtGIST.

**Aims:**

To investigate (i) the diagnostic role of [^68^ Ga]Ga-DOTATATE PET/CT; and, (ii) to investigate the potential of this imaging modality to guide treatment with [^177^Lu]Lu-DOTATATE in patients with wtGIST.

**Methods:**

[^68^ Ga]Ga-DOTATATE PET/CT was performed on 11 patients with confirmed or metastatic wtGIST and one patient with a history of wtGIST and a mediastinal mass suspicious for metastatic wtGIST, who was subsequently diagnosed with a metachronous mediastinal paraganglioma. Tumour expression of somatostatin receptor subtype 2 (SSTR2) using immunohistochemistry was performed on 54 tumour samples including samples from 8/12 (66.6%) patients who took part in the imaging study and 46 tumour samples from individuals not included in the imaging study.

**Results:**

[^68^ Ga]Ga-DOTATATE PET/CT imaging was negative, demonstrating that liver metastases had lower uptake than background liver for nine cases (9/12 cases, 75%) and heterogeneous uptake of somatostatin tracer was noted for two cases (16.6%) of wtGIST. However, [^68^ Ga]Ga-DOTATATE PET/CT demonstrated intense tracer uptake in a synchronous paraganglioma in one case and a metachronous paraganglioma in another case with wtGIST.

**Conclusions:**

Our data suggest that SSTR2 is not a diagnostic or therapeutic target in wtGIST. [^68^ Ga]Ga-DOTATATE PET/CT may have specific diagnostic utility in differentiating wtGIST from other primary tumours such as paraganglioma in patients with sporadic and hereditary forms of wtGIST.

## Introduction

Gastrointestinal stromal tumours (GIST) are the most common mesenchymal tumours of the gastrointestinal tract, and the majority of adult onset GIST, so-called ‘tyrosine kinase mutant’ GIST (TK-mutant GIST), are driven by activating somatic mutations in the *KIT* [1] or *PDGFR*A [2] genes. Wild-type GIST (wtGIST) refers to tumours that are negative for *KIT* and *PDGFRA* gene mutations and account for 15% of adult and 85% of paediatric GIST [3]. wtGIST can be further classified based on the functional status of tumoral succinate dehydrogenase enzyme, as determined by the loss or preservation of SDHB protein expression, following immunohistochemical analysis, as a surrogate marker for a deficient or competent SDH complex [4]. Succinate dehydrogenase competent (cSDH) wtGIST may be associated with an inherited tumour syndrome such as neurofibromatosis type 1 (NF1) [5], or occur sporadically due to somatic mutations in several genes [6, 7] or gene fusion mutations [8]. SDH deficient (dSDH) wtGIST is the most common subcategory of wtGIST [9], and these tumours are driven by inherited germline mutations in one of the *SDHx* genes (*SDHA*/*B*/*C*/*D*) [9] or can occur sporadically following epigenetic silencing of the *SDHC* gene [10]. Patients with dSDH wtGIST are at risk of developing multiple tumour types including phaeochromocytoma/paraganglioma (PPGL) and pulmonary chondroma [10]. Rarely, renal cell carcinoma [11] and pituitary tumours [12] can also develop in patients with germline *SDHx* gene mutations. Molecular profiling of wtGIST has revealed that these tumours share common molecular features with PPGL and other neuroendocrine tumours [13], raising the possibility that established diagnostic tests and therapeutic targets utilised in the clinical management of neuroendocrine tumours, may also have application in the management of wtGIST. Furthermore, the indolent progressive nature of wtGIST is clinically reminiscent of the neuroendocrine tumour phenotype.

While clinical behaviour of wtGIST is generally indolent, there are no effective treatments for patients with more aggressive progressing disease. Management of TK-mutant GIST has been greatly improved by the introduction of the tyrosine kinase inhibitor imatinib which provides an effective standard of care treatment in this setting [14]. Unfortunately, this treatment is ineffective in wtGIST since disease is not driven by tyrosine kinase activity [9]. The availability of a viable theranostic pathway in these tumours would be welcome in this area of unmet clinical need and is being actively explored.

Previous studies have reported high-level expression of the somatostatin receptor 2 (SSTR2) subtype in GIST tumours with unknown mutational status [15] and that GIST tumour sites can be identified with ^111^In-Octreotide scintigraphy in 3/6 cases [16]. Recent case reports evaluating genetically profiled tumours indicate that high SSTR2 receptor overexpression can be found in KIT mutant GIST [17] as well as in dSDH wtGIST [18]. Somatostatin receptor (SSTR) PET/CT has an established role in the localisation of neuroendocrine tumours which highly express SSTR as well as a theranostic role in selecting patients for peptide receptor radionuclide therapy (PRRT) with [^177^Lu]Lu-DOTATATE [19, 20]. Therapy with [^177^Lu]Lu-DOTATATE is highly effective in controlling symptoms and progression of disease in patients with gastroenteropancreatic neuroendocrine tumours and is now approved in many health systems [20]. [^68^ Ga]Ga-DOTATATE PET/CT has emerged as a highly sensitive hybrid SSTR imaging modality for NET as well as for imaging and characterising other tumours with high expression of SSTR such as PPGL [21, 22] and certain mesenchymal tumours [23].

Based on this information, it is possible that SSTR PET/CT may be relevant in the clinical management of patients with wtGIST. This study aims to investigate the diagnostic role of [^68^ Ga]Ga-DOTATATE PET/CT in patients with wtGIST and to assess the potential for theranostic application of the ^177^Lu/^68^ Ga-DOTATATE pair in this setting.

## Methods

### Clinical data collection

Patients were recruited from the National Paediatric and Adult wild-type GIST (PAWS GIST UK), and clinical genetics clinic at Cambridge University Hospital NHS Foundation Trust. Details of clinical phenotype, family history and germline molecular testing results were collated from patient records. All participants provided written informed consent. The study was approved by the East of England South Cambridge Research Ethics Committee (REC ID 14/EE/1059**).**

### *[*^*68*^* Ga]Ga-DOTATATE PET/CT imaging*

Patients were administered 250 (± 10%) MBq of [^68^ Ga]Ga-DOTATATE. Scanning was started after urinary bladder emptying and 60 min after radiopharmaceutical injection on a General Electric Discovery 690 scanner (GE Healthcare, Milwaukee, WI, USA), using 4 min per bed position and low dose CT for attenuation correction and localisation. The ordered subsets expectation maximisation (OSEM) algorithm (2 interations, 24 subsets) was used to reconstruct the emission images with time-of-flight modelling. Emission data were corrected for decay, dead time and random coincidences and normalised for injected dose and patient body weight. Studies were viewed on a Xeleris 4 (GE) workstation for clinical review, and region of interest analysis was performed for quantitative assessment. Maximum standardised uptake values (SUVmax) were recorded for tumour lesions as well as for normal liver. Lesion to liver uptake ratios was calculated for each case. Lesions with an uptake ratio < 1 were considered to have low/no receptor expression, a ratio of 1–2 was considered equivocal, and a ratio > 2 was defined as representing high receptor expression [24]]. All imaging studies were reviewed by experienced nuclear medicine physicians (LA, IM, JB, IH, HC). [^68^ Ga]Ga-DOTATATE PET/CT was performed on twelve patients with wtGIST. DOTATATE measurements were performed on lesions showing solid features on standard of care imaging and/or the registration CT of the [^68^ Ga]Ga-DOTATATE PET/CT which were at least 2 cm or greater in diameter.

### Immunohistochemistry

SSTR2 immunohistochemistry (IHC) was performed on 2 µm sections of paraffin embedded tumour tissue using a rabbit monoclonal anti-SSTR2 antibody (Abcam, catalogue number ab134152) at a dilution of 1:600. Neuroendocrine cells in gastric mucosa, normal adrenal tissue and a well-differentiated pancreatic neuroendocrine tumour were used as a positive control for SSTR2. Adjacent normal tissue was used as an internal positive control. All IHC results were reviewed by an experienced pathologist (OG) and reported using the following scoring system: 0 = no membranous staining; 1 =  < 25% of cells show membranous staining; 2 =  > 25% of cells show membranous staining; 3 =  > 75% of cells show membranous staining. Data outlining GIST-specific immunohistochemistry markers for the 11 GIST cases included in the imaging study are provided in Additional file 1: Table 1.

**Table 1:** Clinical and molecular features of [^68^ Ga]Ga-DOTATATE PET/CT imaging cohort

Case	Sex	Age	Primary tumour site	Metastases	SDH status	Germline mutation	Tumour epi-mutation	Other tumours
001	F	21	Gastric	Liver nodal peritoneal	dSDH	No	*SDHC* epimutation	No
002	M	37	Gastric	Liver, peritoneal	dSDH	*SDHB* c.137G > A, p.(Arg46Gln)	No	Carotid PGL
003	F	31	Gastric	Liver, peritoneal	dSDH	*SDHA* (c.1909 2A > G)	No	No
004	M	39	Gastric	Liver, peritoneal	dSDH	No	No	No
005	F	15	Gastric	Liver, peritoneal	dSDH	*SDHA* c.91C > T p.(Arg31Ter)	Yes	No
006	M	68	Gastric	Liver	dSDH	*SDHD* c.296delT p.(Leu99fs)	No	No
007	M	21	Gastric	Liver	dSDH	*SDHA* c.91C > T p.(Arg31Ter)	No	No
008	F	33	Gastric	Liver	dSDH	No	NA	No
009	M	73	Gastric	Liver nodal	dSDH	Variant of uncertain significance in *SDHA*	No	No
010	M	60	Small bowel	Liver	cSDH	No	No	No
011	M	63	Small bowel	Liver	cSDH	No	No	No
012	F	15	Gastric	Mediastinal mass	dSDH	No	*SDHC* epimutation	Mediastinal mass

NA = not available

### Germline and tumour genetic sequencing

#### Clinical germline DNA sequencing

DNA was extracted from peripheral blood samples according to standard protocols. Next generation sequencing of a clinical gene panel that included *SDHA*, *SDHB*, *SDHC*, *SDHD*, *KIT PDGFRA* and *NF1* was performed at Cambridge University Hospital NHS Foundation Trust using the TrusightOne sequencing panel (Illumina Inc., UK). An average coverage depth of > 20 fold was achieved for 98% of the regions sequenced. Sanger sequencing confirmed all detected variants. Multiple ligation probe analysis (MLPA) was performed for *SDHB*, *SDHC* and *SDHD.*

#### SDHC epimutation analysis

This was performed on DNA extracted from paraffin embedded tumour tissue and adjacent normal tissue using a pyrosequencing method previously described [10].

#### Standard of care imaging

Standard of care imaging was reviewed alongside the [^68^ Ga]Ga-DOTATATE PET/CT for each patient. Eight patients had CT imaging (for two this was the registration CT of the [^68^ Ga]Ga-DOTATATE PET/CT), three patients had MRI, and one patient had CT and MRI at the time of the [^68^ Ga]Ga-DOTATATE PET/CT study. Six patients also had [^18^F]FDG PET/CT studies for comparison.

### Statistical analysis

Statistical analysis was performed using MedCalc (version 18.2.1). A mean and standard deviation was calculated for all continuous variables. An unpaired student t test was employed to investigate differences between groups.

## Results

### *Genotype and clinical phenotype of [*^*68*^* Ga]Ga-DOTATATE PET/CT imaging patient cohort*

Eleven patients with a diagnosis of metastatic wtGIST and one patient with a mediastinal mass and suspected metastatic wtGIST (case 012) were recruited for [^68^ Ga]Ga-DOTATATE PET/CT imaging (Table 1). The mean age at diagnosis was 39 years (range 15–73 years). The liver was the most common site for tumour metastases, which were present in 11/12 patients (92%). One patient (case 002) had a confirmed synchronous carotid paraganglioma. Ten patients had a history of dSDH wtGIST, and two patients had cSDH wtGIST. Five patients (41.6%) had a pathogenic germline *SDHx* variant, one patient had a variant of uncertain significance in the *SDHA* gene, and two patients had a confirmed *SDHC* epimutation. One patient had a dSDH wtGIST but no identifiable germline variant and insufficient tissue available for *SDHC* methylation analysis (case 008) (Table 1).

### Ex vivo* analysis of SSTR2 expression*

Tumour tissue for SSTR2 IHC was performed on 54 tumour samples and this included tumour samples from 8/12 (66.6%) patients who had [^68^ Ga]Ga-DOTATATE PET/CT imaging and 47 tumour samples from individuals who did not have [^68^ Ga]Ga-DOTATATE PET/CT imaging.

**Fig. 1:**
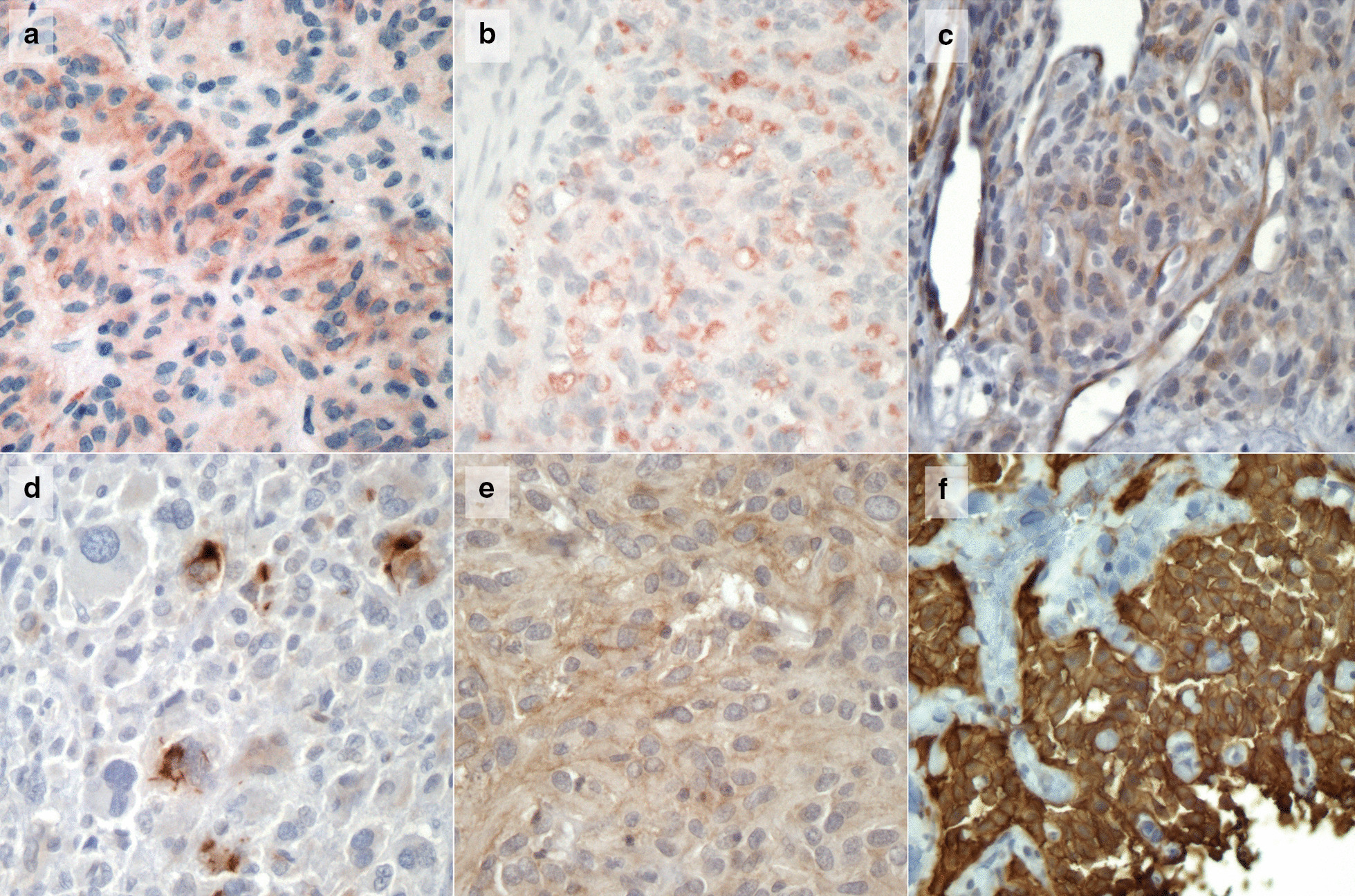
Images **a**–**e** demonstrates negative SSTR2 IHC for cases 005, 006, 007, 009 and aTK mutant GIST case (scores 1, 0, 1, 1 and 2, respectively). Figure F shows SSTR2 immunopositivity in a well-differentiated pancreatic neuroendocrine tumour used as a control (score 3)

SSTR2 immunohistochemistry was performed on tumour samples from eight of the patients who underwent [^68^ Ga]Ga-DOTATATE PET/CT (patient 002, 003, 004, 005, 006, 007, 009 and 010). Single isolated tumour cells showed immunoreactivity in patient 009 (score 1); faint membranous staining (score 1) was seen in patients 005 and 007. Tumours from patients 002, 004, 003, 006 and 010 were negative for SSTR2 and were assigned a score of 0 (Table 2). Of note, a moderate cytoplasmic staining, which we interpreted as non-specific as not located on the exterior cellular membrane (Fig. 1 b), was observed in patient 006.

**Table 2:** [^68^ Ga]Ga-DOTATATE PET/CT scan and IHC findings

Case	SUVmax of most avid lesion	Location	Clinical report	Lesion to liver ratio	SSTR2 IH score
001	3	Liver	Negative	< 1	NA
002	13	Liver	Equivocal (GIST lesion)	2	0
	60	Carotid PGL	Positive (PGL)	> 2.5 (9.6)	NA
003	10	Liver	Equivocal	1.5	0
004	2	Liver	Negative	< 1	0
005	3	Liver	Negative	< 1	1
006	12	Liver	Negative	< 1	0
007	4	Liver	Negative	< 1	1
008	4	Liver	Negative	< 1	NA
009	10	Liver, nodal	Negative	< 1	1
010	2.5	Liver	Negative	< 1	0
011	2.8	Liver	Negative	< 1	NA
012	45	Mediastinal mass	Positive	> 2.5 [9]	NA
	40	Mediastinal node	Positive	> 2.5 [8]	

NA = not available

**Fig. 2:**
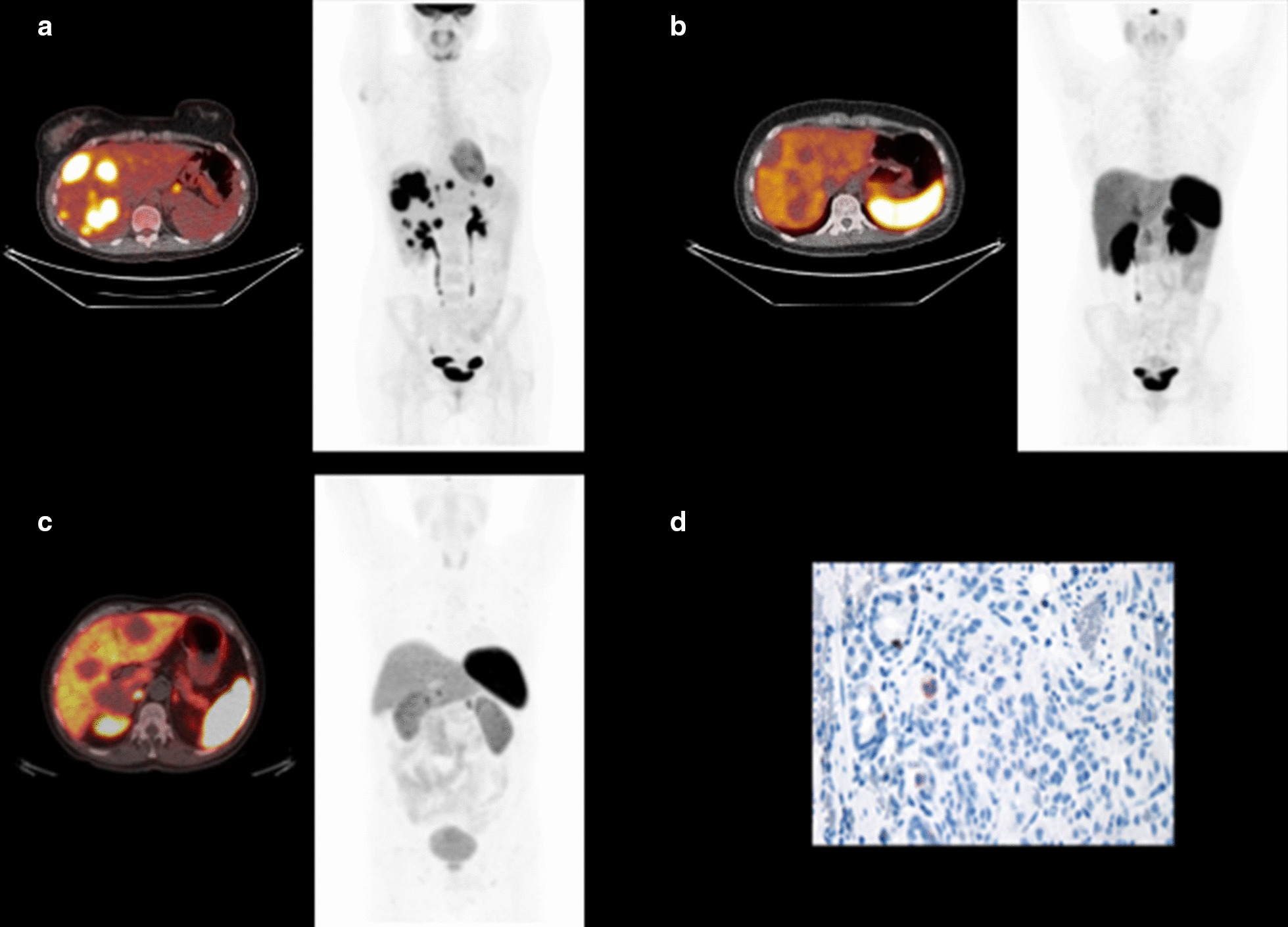
Panel A shows an axial fused [^18^F]FDG PET/CT image of the liver with a maximum intensity projection (MIP) image demonstrating highly FDG-avid liver and nodal metastases in case 001. Panel B shows the corresponding (case 001) trans-axial fused image of the [^68^ Ga]Ga-DOTATATE PET/CT and MIP image demonstrating low tracer uptake in the liver metastases (SUVmax 3) compared to background liver. Panel C, fused transaxial [^68^ Ga]Ga-DOTATATE PET/CT image and corresponding MIP image showing non-avid liver metastases in case 010 (SUVmax 2.5). Panel D shows negative SSTR2 expression of a liver metastasis on IHC for case 010

The 46 tumour samples from individuals who did not have [^68^ Ga]Ga-DOTATATE PET/CT imaging included; 14 dSDH wtGIST, 9 cSDH wtGIST and 23 TK-mutant GIST. The mean SSTR2 expression score was 0.73 for the dSDH wtGIST (8/14 had a score of 0, 3/14 had a score of 1, one case had a score of 2 and two cases had a score of 3) versus 0.22 in the cSDH wtGIST (7/9 had a score of 0 and 2/9 had a score of 1) and 0.22 in the TK-mutant GIST (19/23 had a score of 0, 3/23 had a score of 1 and one case had a score of 2). The median score was 0 for each molecular subgroup of GIST indicating low or absent expression of SSTR2 in the tumour tissue in all GIST tumours reviewed. No significant difference in the mean SSTR2 expression score was demonstrated between molecular sub-groups of GIST (*p* = 0.1) (Fig. 2).

### In vivo* analysis of SSTR2 expression using [*^*68*^* Ga]Ga-DOTATATE PET/CT imaging*

High-quality [^68^ Ga]Ga-DOTATATE PET/CT imaging was obtained for all twelve patients included in this study. Imaging findings are summarised in Table 2. The liver was the most common site of wtGIST metastases in this series (11/12 cases, 91.6%). Measured SUVmax values of identified wtGIST metastases were highest in liver lesions due to high normal background. The mean size of the liver lesions from which SUVmax was 4.6 cm (range 2.1 to 11 cm). The average SUV max for the most avid GIST lesion (one lesion per patient) was 6 (range 2–13), and the average lesion to liver ratio for a presumed GIST lesion was 0.7 (range 0.2 to 2). In most instances (8/12 cases, 75%), metastases showed lower uptake than background liver (lesion to liver uptake ratio < 1) and [^68^ Ga]Ga-DOTATATE PET/CT imaging was reported as negative for high SSTR expression.

**Fig. 3:**
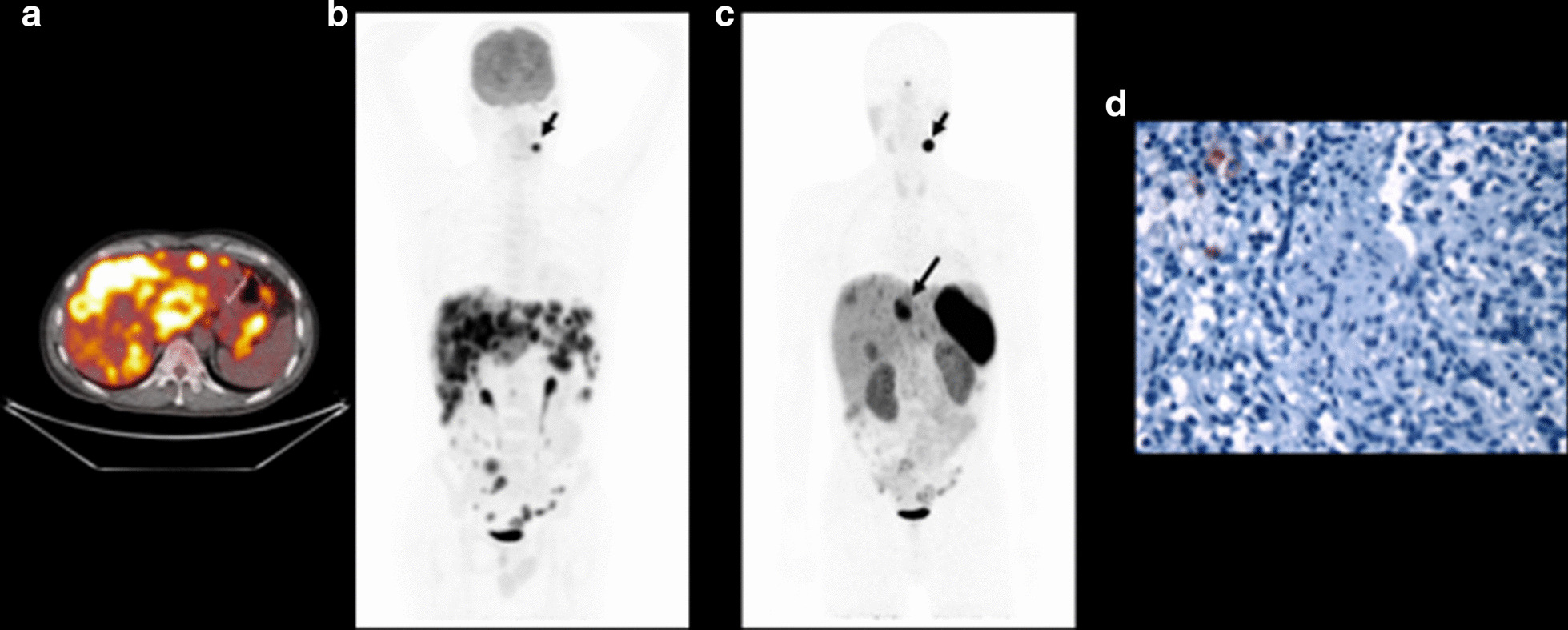
Case 002. Panel A, transaxial fused [^18^F]FDG PET/CT image of the liver and MIP image demonstrating FDG-avid liver, peritoneal and nodal metastases as well as a synchronous left sided carotid paraganglioma (small arrow). Panel B, [^68^ Ga]Ga-DOTATATE PET/CT MIP image, demonstrating that few liver metastases show uptake higher than background liver (SUVmax 13, large arrow, vs 6.5 for background liver). It should be noted that not all liver lesions were biopsied so it remains unclear if all liver lesions were metastatic wtGIST deposits or if the carotid paraganglioma was also metastatic to the liver. The left sided carotid paraganglioma shows very intense uptake of the somatostatin analogue (SUVmax 60, small arrow). Panel C, negative SSTR2 expression on IHC in a biopsied dSDH wtGIST liver metastasis from case 002

In four cases, lesions showed [^68^ Ga]Ga-DOTATATE uptake which was equal to or above that of background liver. In one case (case 012), the mediastinal mass suspicious for a metastatic recurrence of a previous wtGIST was subsequently confirmed as a mediastinal paraganglioma explaining the high [^68^ Ga]Ga-DOTATATE uptake in this lesion. Similarly for case 002, where several liver lesions showed uptake slightly higher than liver, this patient had a synchronous carotid paraganglioma, and therefore, we cannot be certain that the liver metastases were all related to the wtGIST and not the co-existing paraganglioma as repeat biopsy was not performed and the patient is now deceased (Fig. 3). Heterogeneous tracer uptake was demonstrated for two cases; case 003, in whom very few of the numerous liver lesions and a left gastric node demonstrated uptake slightly higher than liver; case 009, where a few peritoneal nodules showed [^68^ Ga]Ga-DOTATATE uptake comparable to liver, but multiple disease deposits showing no measurable uptake were documented in liver and abdominal nodes. Of note, anatomical imaging did not show significant necrosis and/or cystic features in any of the patients/lesions analysed to justify the low tracer uptake.

**Fig. 4:**
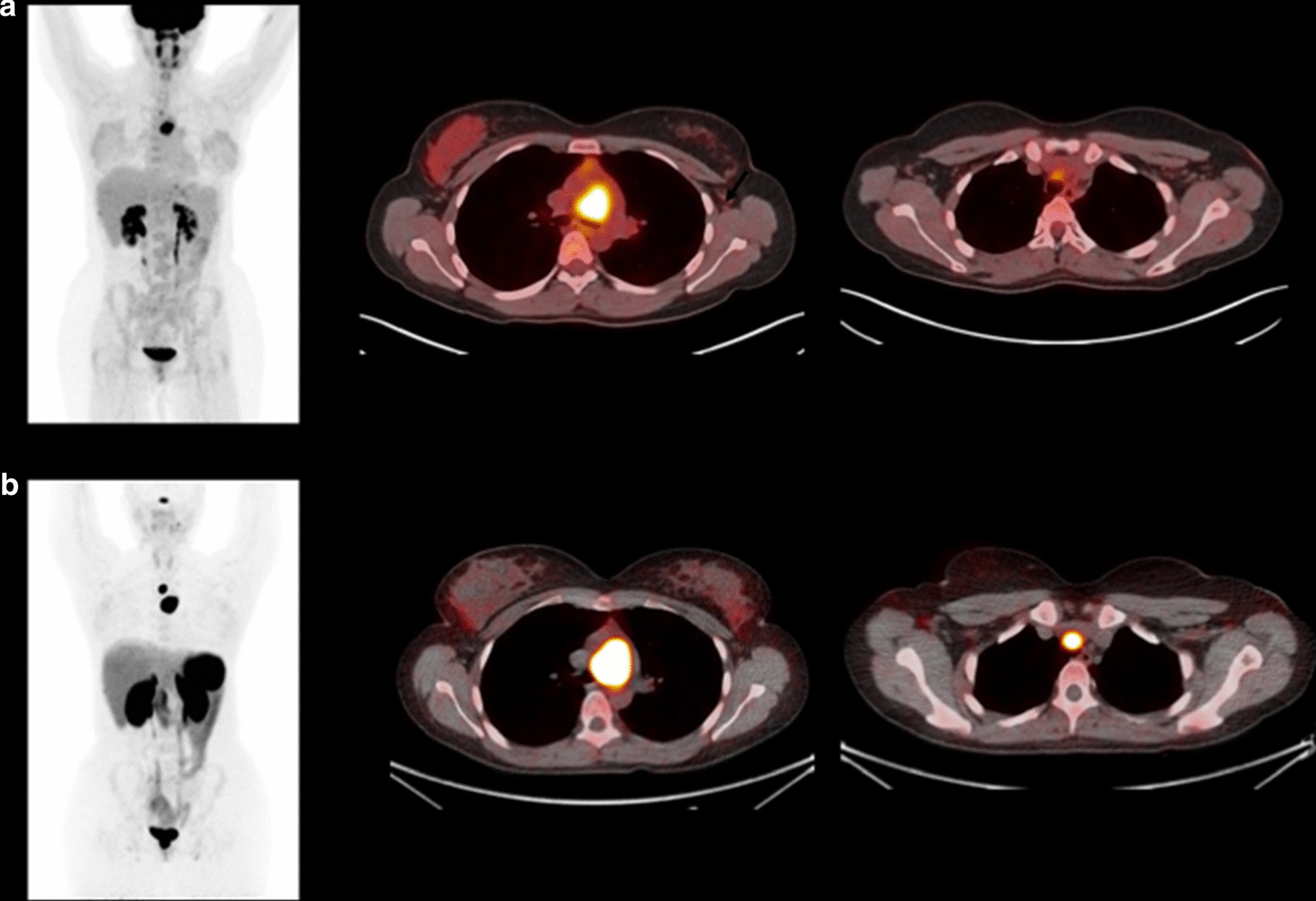
Case 012. Panel A, [^18^F]FDG PET/CT MIP image and fused transaxial views showing a highly avid mediastinal mass and low-level uptake of an adjacent lymph node (C). This imaging was performed as part of surveillance for disease recurrence given a background of a resected wild-type GIST, and the mediastinal mass was a presumed recurrence of this earlier tumour. Panel B, ^68^ Ga-DOTATATE PET/CT, same views as above. Very intense uptake in the mediastinal mass (SUVmax 45) and adjacent lymph node (SUVmax 40). This patient had normal plasma metanephrine and 3-methoxytyramine values on biochemical testing. Biopsy confirmed a non-secreting mediastinal dSDH paraganglioma case 012

A negative or equivocal scan report for 11/12 patients with metastatic wtGIST correlated with the low or absent expression of SSTR2 ex vivo (Table 2). High [^68^ Ga]Ga-DOTATATE avidity (> 2 times normal liver) was only noted in the synchronous or metachronous PPGL lesions from case 2 and 12 (Fig. 4).

## Discussion

To our knowledge, this is the first study to investigate the clinical utility of [^68^ Ga]Ga-DOTATATE PET/CT in patients with wtGIST. In this study, SSTR2 expression was demonstrated to be low or absent for each of the 11 cases of metastatic wtGIST analysed using [^68^ Ga]Ga-DOTATATE PET/CT and in tumour samples from 8/12 patients using SSTR2 IHC. Notably, SSTR2 expression was also found to be low or absent in an additional 46 tumour samples, including 23 TK-mutant GIST and an additional 23 wtGIST samples. A previous study investigating the expression of SSTR1-5 in TK-mutant GIST demonstrated expression of SSTR1-5 using both quantitative real-time polymerase chain reaction (qPCR) and IHC. In this earlier study, the expression of SSTR2 was lower than other SSTR subtypes and no wtGISTs were included in the analysis [16]. Zhao et al. investigated SSTR1-5 expression in GIST using qPCR and IHC in approximately 500 tumour samples and high expression levels of both SSTR1 and SSTR2 were noted; however, details regarding the molecular classification of the GIST samples were not provided [15]. While the additional anecdotal evidence from imaging studies [17, 18] encouraged us to pursue the possibility of a SSTR-2 targeting theragnostic strategy in this setting, the data obtained indicate that, at least in the case of wtGIST, this approach is not viable.

Alternative molecular targets are being investigated for radionuclide-based theranostics in GIST such as the gastrin releasing peptide/bombesin receptor (GRPR). PET imaging with a ^68^ Ga-labelled GRPR agonist identified lesions in 7/17 patients with GIST [25]. More recently, a ^68^ Ga-labelled GRPR antagonist (NeoBomb1) has been shown to have high selective binding in GIST derived cell lines [26]. These findings have led to a small phase 1/2a study assessing the safety and efficacy of this ligand for PET imaging of oligometastatic GIST [27], with the aim of developing a theranostic pathway based on this targeting strategy.

One indication for [^68^ Ga]Ga-DOTATATE PET/CT is to confirm the diagnosis in patients with anatomic lesions that are suspicious for NET on conventional imaging [28]. The ability to utilise [^68^ Ga]Ga-DOTATATE PET/CT imaging as a virtual biopsy for NET is dependent on a good knowledge of the physiological uptake of [^68^ Ga]Ga-DOTATATE in normal tissue and recognising possibility of high tracer uptake in non-neuroendocrine tumours [29]. A previous case study using [^68^ Ga]Ga-DOTATATE PET/CT imaging in a patient with a suspected metastatic dSDH wtGIST demonstrated high SSTR2 tumour expression; however, this case had a synchronous parapharyngeal paraganglioma and the authors of this study rightfully acknowledged that the metastatic lesions identified on [^68^ Ga]Ga-DOTATATE PET/CT imaging may relate to the PPGL rather than wtGIST [18].

The conventional radiological measurement of tumour response including uni- or bidimensional changes in tumour size (response evaluation criteria in solid tumours, RECIST) is routinely applied in clinical practice for both GIST and wtGIST using CT or MRI. The benefit of evaluating metabolic response using [^18^F]FDG PET/CT is well demonstrated in *KIT-* or *PDGFRA-* mutated GIST tumours following treatment with the tyrosine kinase inhibitor; imatinib, [30] as mesenchymal tumours such as GIST rarely demonstrate a reduction in size despite therapeutic response. As the majority of wtGIST are SDH-deficient, these tumours exhibit upregulation of hexokinase receptors [31] and increased ^18^F-FDG uptake, and therefore, it is likely that this may mitigate any benefit of ^18^F-FDG PET/CT in determining therapeutic response to tyrosine kinase inhibitors in wtGIST, but ^18^F-FDG PET/CT remains a sensitive imaging modality for SDH deficient tumours [31] although it lacks the ability to differentiate between tumour types (e.g. PGL versus wtGIST).

Patients with dSDH wtGIST due to a germline variant in *SDHx* or *SDHC* epimutations are at risk of multiple tumours including multifocal PPGL, pulmonary chondroma and renal cell carcinoma. In this study, case 002 had a metastatic dSDH wtGIST and a synchronous carotid paraganglioma. Notably, a few liver metastases demonstrated uptake higher than background liver (SUVmax 13) and the carotid paraganglioma demonstrated very high somatostatin tracer uptake. It is worth noting that not all liver lesions were biopsied, and therefore, it is not certain if some of the liver lesions may have been metastatic deposits from the carotid paraganglioma rather than the dSDH wtGIST. Furthermore, patients with cSDH wtGIST associated with NF1 are also predisposed to the development of multiple tumours over their lifetime. Notably, the location of PPGL in patients with germline *SDHx* mutations or *SDHC* epimutations can be anywhere from the skull base to the pelvis and dSDH PPGL also have a high malignant potential [32], increasing the risk for synchronous malignant primary tumours in one individual. Therefore, as [^18^F]FDG PET/CT lacks the ability to differentiate between at risk tumour types in patients with SDHx mutations, there is an unmet need for sensitive and specific imaging modalities which are able to distinguish PPGL from wtGIST in this patient population in order to avoid multiple biopsies and inform appropriate management with proper use of molecular imaging.

In this study, [^68^ Ga]Ga-DOTATATE PET/CT imaging was negative for nine patients and equivocal for two patients with metastatic wtGIST. However, the uptake level in a synchronous carotid PPGL (SUVmax 60) in case 002 and a metachronous mediastinal PPGL in case 012 (SUVmax 45) was almost ten-fold higher than liver reference in these individuals and 9–tenfold higher than the average uptake seen in metastases of 11 cases of wtGIST (mean SUVmax 6). These results suggest that [^68^ Ga]Ga-DOTATATE PET/CT imaging may have specific utility for differentiating PPGL from wtGIST in clinical practice in patients with a genetic predisposition to develop both tumour types.

## Conclusions

Our results address conflicting reports in the literature on the role of SSTR2 in wtGIST and indicate that this receptor system is not a viable diagnostic or therapeutic target. We show that [^68^ Ga]Ga-DOTATATE PET/CT may have an important diagnostic role in identifying and differentiating PPGL lesions from metastatic wtGIST in patients carrying genetic mutations predisposing to both conditions.

## Supplementary information

**Additonal files 1**. Supplementary Table 1: GIST specific immunohistochemical markers.

## Data Availability

Available on request.

## References

[1] Hirota S, Isozaki K, Moriyama Y, Hashimoto K, Nishida T, Ishiguro S (1998). Gain-of-function mutations of c-kit in human gastrointestinal stromal tumors. Science.

[2] Heinrich MC, Corless CL, Duensing A, McGreevey L, Chen C-J, Joseph N (2003). PDGFRA activating mutations in gastrointestinal stromal tumors. Science.

[3] Janeway KA, Albritton KH, Van Den Abbeele AD, D’Amato GZ, Pedrazzoli P, Siena S (2009). Sunitinib treatment in pediatric patients with advanced GIST following failure of imatinib. Pediatr Blood Cancer.

[4] Papathomas TG, Oudijk L, Persu A, Gill AJ, van Nederveen F, Tischler AS (2015). SDHB/SDHA immunohistochemistry in pheochromocytomas and paragangliomas: a multicenter interobserver variation analysis using virtual microscopy: a multinational study of the european network for the study of adrenal tumors (ENS@T). Mod Pathol.

[5] Gasparotto D, Rossi S, Polano M, Tamborini E, Lorenzetto E, Sbaraglia M (2017). Quadruple-negative GIST is a sentinel for unrecognized neurofibromatosis type 1 syndrome. Clin Cancer Res.

[6] Pantaleo MA, Nannini M, Corless CL, Heinrich MC (2015). Quadruple wild-type (WT) GIST: defining the subset of GIST that lacks abnormalities of KIT, PDGFRA, SDH, or RAS signaling pathways. Cancer Med.

[7] Nannini M, Urbini M, Astolfi A, Biasco G, Pantaleo MA (2017). The progressive fragmentation of the KIT/PDGFRA wild-type (WT) gastrointestinal stromal tumors (GIST). J Transl Med.

[8] Brenca M, Rossi S, Polano M, Gasparotto D, Zanatta L, Racanelli D (2016). Transcriptome sequencing identifies ETV6-NTRK3 as a gene fusion involved in GIST. J Pathol.

[9] Boikos SA, Pappo AS, Killian JK, LaQuaglia MP, Weldon CB, George S (2016). Molecular SUBTYPES of KIT/PDGFRA wild-type gastrointestinal stromal tumors. JAMA Oncol.

[10] Casey RT, Ten Hoopen R, Ochoa E, Challis BG, Whitworth J, Smith PS (2019). SDHC epi-mutation testing in gastrointestinal stromal tumours and related tumours in clinical practice. Sci Rep.

[11] Casey RT, Warren AY, Martin JE, Challis BG, Rattenberry E, Whitworth J (2017). Clinical and molecular features of renal and pheochromocytoma/paraganglioma tumor association syndrome (RAPTAS): case series and literature review. J Clin Endocrinol Metab.

[12] Xekouki P, Stratakis CA (2012). Succinate dehydrogenase (SDHx) mutations in pituitary tumors: could this be a new role for mitochondrial complex II and/or Krebs cycle defects?. Endocr Relat Cancer.

[13] Pantaleo MA, Urbini M, Indio V, Ravegnini G, Nannini M, De Luca M (2017). Genome-wide analysis identifies MEN1 and MAX mutations and a neuroendocrine-like molecular heterogeneity in quadruple WT GIST. Mol Cancer Res.

[14] Joensuu H, Roberts PJ, Sarlomo-Rikala M, Andersson LC, Tervahartiala P, Tuveson D (2001). Effect of the tyrosine kinase inhibitor STI571 in a patient with a metastatic gastrointestinal stromal tumor. N Engl J Med.

[15] Zhao W-Y, Zhuang C, Xu J, Wang M, Zhang Z-Z, Tu L (2014). Somatostatin receptors in gastrointestinal stromal tumors: new prognostic biomarker and potential therapeutic strategy. Am J Transl Res.

[16] Arne G, Nilsson B, Dalmo J, Kristiansson E, Arvidsson Y, Forssell-Aronsson E (2013). Gastrointestinal stromal tumors (GISTs) express somatostatin receptors and bind radiolabeled somatostatin analogs. Acta Oncol.

[17] Braat AJAT, Goldschmeding R, Brosens LAA, Vriens MR, de Keizer B (2017). Gastrointestinal stromal tumour detection with somatostatin receptor imaging, 68Ga-HA-DOTATATE PET–CT. Lancet Oncol.

[18] Loaiza-Bonilla A, Bonilla-Reyes PA (2017). Somatostatin receptor avidity in gastrointestinal stromal tumors: theranostic implications of Gallium-68 scan and eligibility for peptide receptor radionuclide therapy. Cureus.

[19] Jaïs P, Terris B, Ruszniewski P, LeRomancer M, Reyl-Desmars F, Vissuzaine C (1997). Somatostatin receptor subtype gene expression in human endocrine gastroentero-pancreatic tumours. Eur J Clin Invest.

[20] Strosberg J, El-Haddad G, Wolin E, Hendifar A, Yao J, Chasen B (2017). Phase 3 trial of 177Lu-dotatate for midgut neuroendocrine tumors. N Engl J Med.

[21] Jha A, Ling A, Millo C, Gupta G, Viana B, Lin FI (2018). Superiority of 68Ga-DOTATATE over 18F-FDG and anatomic imaging in the detection of succinate dehydrogenase mutation (SDHx )-related pheochromocytoma and paraganglioma in the pediatric population. Eur J Nucl Med Mol Imaging.

[22] Kong G, Schenberg T, Yates CJ, Trainer A, Sachithanandan N, Iravani A (2019). The role of 68Ga-DOTA-octreotate PET/CT in follow-up of sdh-associated pheochromocytoma and paraganglioma. J Clin Endocrinol Metab.

[23] Neuzillet C, de Mestier L, Rousseau B, Mir O, Hebbar M, Kocher HM (2018). Unravelling the pharmacologic opportunities and future directions for targeted therapies in gastro-intestinal cancers part 2: Neuroendocrine tumours, hepatocellular carcinoma, and gastro-intestinal stromal tumours. Pharmacol Ther.

[24] Ilias I, Chen CC, Carrasquillo JA, Whatley M, Ling A, Lazurova I (2008). Comparison of 6–18F-fluorodopamine PET with 123I-metaiodobenzylguanidine and 111In-pentetreotide scintigraphy in localization of nonmetastatic and metastatic pheochromocytoma. J Nucl Med.

[25] Dimitrakopoulou-Strauss A, Hohenberger P, Haberkorn U, Mäcke HR, Eisenhut M, Strauss LG (2007). 68Ga-labeled bombesin studies in patients with gastrointestinal stromal tumors: comparison with 18F-FDG. J Nucl Med.

[26] Paulmichl A, Summer D, Manzl C, Rangger C, Orlandi F, Niedermoser S (2016). Targeting gastrointestinal stromal tumor with 68 Ga-labeled peptides: an in vitro study on gastrointestinal stromal tumor-cell lines. Cancer Biother Radiopharm.

[27] Gruber L, Jimenez-Franco LD, Decristoforo C, Uprimny C, Glatting G, Hohenberger P, et al. MITIGATE-NeoBOMB1, a Phase I/IIa Study to Evaluate Safety, Pharmacokinetics and Preliminary Imaging of 68Ga-NeoBOMB1, a Gastrin-releasing Peptide Receptor Antagonist, in GIST Patients. J Nucl Med. 2020. 10.2967/jnumed.119.238808 32332143

[28] Subramaniam RM, Bradshaw ML, Lewis K, Pinho D, Shah C, Walker RC (2018). ACR practice parameter for the performance of gallium-68 DOTATATE PET/CT for neuroendocrine tumors. Clin Nucl Med.

[29] Kuyumcu S, Özkan ZG, Sanli Y, Yilmaz E, Mudun A, Adalet I (2013). Physiological and tumoral uptake of 68Ga-DOTATATE: standardized uptake values and challenges in interpretation. Ann Nucl Med.

[30] Demetri GD, von Mehren M, Blanke CD, Van den Abbeele AD, Eisenberg B, Roberts PJ (2002). Efficacy and safety of imatinib mesylate in advanced gastrointestinal stromal tumors. N Engl J Med.

[31] van Berkel A, Rao JU, Kusters B, Demir T, Visser E, Mensenkamp AR (2014). Correlation between in vivo 18F-FDG PET and immunohistochemical markers of glucose uptake and metabolism in pheochromocytoma and paraganglioma. J Nucl Med.

[32] Gimenez-Roqueplo A-P, Favier J, Rustin P, Rieubland C, Crespin M, Nau V (2003). Mutations in the SDHB gene are associated with extra-adrenal and/or malignant phaeochromocytomas. Cancer Res.

[33] van Berkel A, Rao JU, Kusters B, Demir T, Visser E, Mensenkamp AR (2014). Correlation Between In Vivo 18F-FDG PET and Immunohistochemical Markers of Glucose Uptake and Metabolism in Pheochromocytoma and Paraganglioma. J Nucl Med.

