# Supplementary material for: The role of [68 Ga]Ga-DOTATATE PET/CT in wild-type KIT/PDGFRA gastrointestinal stromal tumours (GIST)
Source: EJNMMI Res. 2021 Jan 14;11:5. doi: 10.1186/s13550-021-00747-0 (PMC7809083; doi:10.1186/s13550-021-00747-0)
Supplement: Supplementary file 1 — Additonal files 1. Supplementary Table 1: GIST specific immunohistochemical markers. [file 13550_2021_747_MOESM1_ESM.docx]

Supplementary Table 1: GIST specific immunohistochemical markers

| **Case** | **Histological type** | **Mitotic count/5mm2** | **CD117** | **DOG1** | **CD34** |
| --- | --- | --- | --- | --- | --- |
| 001 | Mixed epithelioid | 2 | Strong | Strong, diffuse | NA |
| 002 | Epithelioid | 4 | Patchy | Strong, diffuse | Positive |
| 003 | Spindle | 4 | Strong | Strong | Positive |
| 004 | Epithelioid | 10 | Strong | Strong | NA |
| 005 | Epithelioid | 6 | Strong | NA | Positive |
| 006 | Mixed epithelioid | 1 | Strong | Strong | NA |
| 007 | Spindle | 3 | NA | NA | NA |
| 008 | Spindle | 2 | Strong | Strong | Strong |
| 009 | Mixed epithelioid | 18 | Strong | Strong | Strong |
| 010 | Mixed epithelioid | 12 | Strong | Strong | NA |
| 011 | NA | NA | Strong | Strong | NA |

NA= not available
